# Prenatal Screening for Developmental Displacement of the Hip: The BUDDHA (Pre-Birth Ultrasound for Developmental Displacement of the Hip Assessment) Study

**DOI:** 10.3390/diagnostics11050868

**Published:** 2021-05-12

**Authors:** Elena Contro, Laura Larcher, Jacopo Lenzi, Arianna Benfenati, Giulia Massinissa Magini, Giulia Galeati, Maria Terrone, Silvia Galletti, Santo Arcuri, Anna Seidenari, Antonio Farina

**Affiliations:** 1 Department of Medicine and Surgery, Division of Obstetrics and Prenatal Medicine, IRCCS Sant’Orsola-Malpighi, University of Bologna, 40138 Bologna, Italy; laura.larcher@studio.unibo.it (L.L.); arianna.benfenati@gmail.com (A.B.); giulia.galeati3@studio.unibo.it (G.G.); maria_terrone@live.it (M.T.); annaseidenari@gmail.com (A.S.); antonio.farina@unibo.it (A.F.); 2 Section of Hygiene, Public Health and Medical Statistics, Department of Biomedical and Neuromotor Sciences, Alma Mater Studiorum, University of Bologna, 40138 Bologna, Italy; jacopo.lenzi2@unibo.it; 3 Neonatology Unit, Department of Medical and Surgical Sciences, IRCCS St. Orsola-Malpighi, University of Bologna, 40138 Bologna, Italy; gmassinissa@virgilio.it (G.M.M.); silvia.galletti4@unibo.it (S.G.); santo.arcuri@unibo.it (S.A.)

**Keywords:** alpha and beta angles, Graf technique, antenatal ultrasound, coxofemoral joint, developmental dysplasia of the hip, prenatal screening

## Abstract

Background: developmental dysplasia of the hip has an incidence of 3–5 out of 1000 children. Currently, only postnatal screening is available. Objective: to test the feasibility of a method based on Graf technique application at antenatal ultrasound in assessing the normal development of the hip in unselected term fetuses. Methods: a prospective cohort study in a single university tertiary hospital from January 2017 to January 2020. Single uncomplicated term pregnancies (37–40 weeks) attending our center for routine ultrasound were consecutively recruited for the purpose of the study. A 3D volume acquisition was launched on the coxofemoral joint of the fetus by a single expert operator, and offline analysis was then performed in the multiplanar mode by two operators (blinded to each other analysis) in order to measure the alpha and beta angles according to our modified Graf technique. Intra- and inter-observer variations were calculated. Reference charts for normal values of both angles were produced. Postnatal ultrasound was then performed to measure the Graf angles in newborns, confirming a normal development of the hip. Results: in the study period, 433 uncomplicated term pregnancies underwent 3D ultrasound for the assessment of the fetal hip. One case was subsequently excluded because of confirmed postnatal diagnosis of developmental dysplasia of the hip. The measurement of our modified Graf angles was feasible at prenatal ultrasound with a good reproducibility. The inter-rater and intra-rater reliability of both angles was substantial. Reference charts for normal values of both angles were produced. Conclusions: the evaluation of the coxofemoral joint in fetuses at term of gestation has never been attempted before. The Graf technique application, currently employed at postnatal ultrasound, may also be adapted to prenatal ultrasound with a substantial reproducibility. However, there was no evidence of a linear relationship between prenatal and postnatal alpha angles and beta angles. Further research is needed to establish if developmental dysplasia of the hip could be diagnosed antenatally.

## 1. Introduction

Developmental dysplasia of the hip is a spectrum of conditions that includes a mechanical instability of the hip joint due to an anomalous development of the acetabulum and proximal femur. The incidence of developmental dysplasia of the hip demonstrates high variability in literature due to the ambiguity of criteria for defining pathological hips, geographical differences, genetic predisposition, and cultural practices. The most recognized incidence of dislocatable hips and hips with severe or persistent dysplasia is 3 to 5 per 1000 children [1,2,3]. However, Barrera et al. [4] reported an incidence of developmental dysplasia of the hip up to 20 per 1000 live births.

The etiology of developmental dysplasia of the hip is multifactorial, but established risk factors are breech presentation, female gender, positive family history, and being first born [5,6]. Breech presentation has been recognized as the most important antenatal non-genetic risk factor for developmental dysplasia of the hip and increases the risk of this condition up to 24 times when compared with cephalic presentation [7]. Developmental dysplasia of the hip is diagnosed in 2–27% of newborns delivered in breech presentation, in particular those in frank breech presentation (with extended knees and feet close to the baby face) because this position in utero leads to hip extension and adduction, that, if kept for prolonged time, can affect the normal development of the coxofemoral articulation [8]. Other mechanical factors that have been associated with an increased risk for developmental dysplasia of the hip are twin pregnancy, oligohydramnios, and large for gestational age fetus [9,10]. All these conditions affect fetal movements in utero and can force the coxofemoral articulation to a prolonged hip extension and adduction, that can lead to an abnormal development of this articulation.

The left side is involved in 60% of the children, the right side in 20%, whereas 20% have bilateral involvement [9]. This is probably due to the fetal position in utero; indeed, LOA (left occiput anterior position) is the most common fetal position near term. In this position, the left coxofemoral articulation of the fetus is forced against the spinal column of the mother and the possibility of fetal movement is limited.

Early diagnosis of developmental dysplasia of the hip has been demonstrated to improve the outcome of affected babies. Residual sequelae of this condition are one of the leading causes of early hip osteoarthritis in adulthood [11]. Indeed, if left untreated, developmental dysplasia of the hip can lead to secondary damage of the head of the femur, articular cartilage disruption, and different degrees of movement compromise [9]. On the contrary, early recognition of developmental dysplasia of the hip allows for a complete correction of this condition without any clinical sequelae. Conservative treatment is possible within the first six months of life; after this interval of time, only more invasive therapies (requiring immobilization) or surgery are available, and a complete correction is not achieved in most cases. First-line treatment includes non-invasive techniques (such as Pavlik Harness) that counteract hip adduction and keep the hip articulation reduced in abduction. A six-week treatment before six months of life has been demonstrated to solve the hip instability in 99% of cases [10]. Therefore, the early detection of developmental dysplasia of the hip has become extremely important in preventive medicine. Different clinical and ultrasound techniques have been proposed for the early diagnosis of this condition. The Ortolani manouvres are commonly employed in the clinical examination of newborns, yet the sensitivity of this technique is quite low and only more severe cases are identified with this method [12]. The Graf technique is an ultrasound technique firstly introduced in Austria in 1980 [13]. This method is now universally employed to diagnose developmental dysplasia of the hip in infants. It has been demonstrated to have a better sensitivity and specificity when compared to other techniques, with a good intra- and inter-observer reproducibility [14]. Even if different screening protocols have been proposed to spot developmental dysplasia of the hip in infants, most countries have abandoned universal ultrasound screening programs due to lack of resources, and therefore a dedicated ultrasound examination is offered only to high-risk patients or to those with clinical suspicion. In other countries, no ultrasound examination is available at all. As a result of this, the diagnosis of developmental dysplasia of the hip is late in many cases, with limited chance to obtain a complete functional recovery [9].

An antenatal identification of fetuses with developmental dysplasia of the hip, or with an immaturity of the hip, that are at increased risk of developing this disorder after birth, would be useful in clinical practice. To our knowledge, no case of prenatal diagnosis of this pathology has been reported in literature and no normal range of fetal Graf angles are available for fetuses at term of gestation.

The objective of this study was to test the feasibility of antenatal ultrasound, using the postnatal technique based on Graf angles, to assess the normal development of the hip in unselected term fetuses.

## 2. Materials and Methods

### 2.1. Study Design

This was a prospective cohort study. Before the experiment started, an institutional review board approval was obtained. From January 2017 to January 2020, all eligible single uncomplicated term pregnancies (37–40 weeks) attending our center for routine ultrasound were consecutively recruited for the purpose of the study. A 3D volume of the fetal hip was obtained in all cases when possible both fetal hips were insonated. Only the best volume acquisition was used for angles calculation in each fetus, irrespective of laterality.

### 2.2. Patient Involvement

Inclusion criteria were age >18 or <48 years, gestational age from 37 week to 40 weeks, and single pregnancy in cephalic presentation or in breech presentation.

Patients were excluded if any of the following conditions were present: evidence or suspicion of fetal structural or chromosomal abnormality, infectious diseases in pregnancy, twin pregnancy, maternal obesity (BMI > 30 kg/m^2^). Finally, cases with confirmed postanatal diagnosis of DDH were excluded from statistical analysis.

### 2.3. Intervention

A 3D ultrasound volume of the coxofemoral articulation of the fetus was acquired transabdominally using a Voluson E8 (GE, Milan, Italy) equipped with a multifrequency probe.

**Figure 1:**
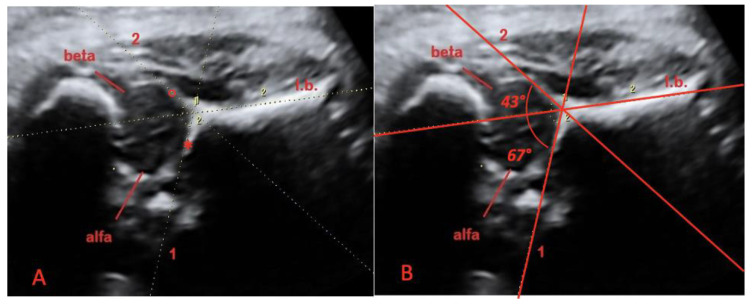
(**A**) Midsagittal plane of the coxofemoral joint, obtained with 3D ultrasound, in a fetus at 37 weeks of gestation: the “Y” sign. The iliac bone (I.b.), the labrum acetabular (°) and the lower edge of ossified ilium (*). (**B**) Midsagittal plane of coxofemoral joint, obtained with 3D ultrasound, in a fetus at 37 weeks of gestation: the “Y” sign. The alpha angle (“bone angle”) is the angle formed by a line tangential to the horizontal iliac wing (I.b.) and a line tangential to bone roof (*). The beta angle (“cartilage angle”) is the angle formed by a line tangential to the horizontal iliac wing (I.b.) and a line tangential to cartilaginous roof (°).

The starting plane for 3D insonation was the midsagittal plane of coxofemoral joint; the “Y” sign was then visualized (Figure 1). When it was possible, we acquired both coxofemoral articulations of the fetus; if it was not possible, only the more proximal articulation was acquired.

All examinations were performed by a single expert operator. The volume dataset of the coxofemoral articulation was acquired and then analyzed offline in the multiplanar mode by two operators (blinded to each other analysis) using the static volume contrast imaging (VCI) mode of the 4D view software (9.0 version, GE, Milan, Italy). The 3D image was adjusted to achieve the midsagittal view of the coxofemoral articulation in the A-plane (“Y sign”), with the reference dot positioned in the middle of the iliac bone, at the level of the sling opening. On the magnified A plane, the alpha and beta angles were calculated according to our modified Graf technique [15,16]. The alpha angle was defined as the angle formed by a line tangential to the horizontal iliac wing and a line tangential to bone roof (Figure 1); this is called the “bone angle”. The alpha angle was assumed as a measure at rest of the depth of the bony acetabulum. The beta angle was defined as the angle formed by a line tangential to the horizontal iliac wing and a line tangential to cartilaginous roof (Figure 1); this is called the “cartilage angle”. The beta angle was assumed as a quantitative measure at rest that that shows coverage of the femoral head by the cartilaginous acetabulum. Each angle was measured twice by two independent operators and for each of the above-mentioned measurements, and intra-observer and inter-observer reproducibility were calculated.

Offline volume analysis by either examiner never exceeded 5 min for each single dataset and led to calculation of both alpha and beta angles in all cases. All neonates underwent a postnatal ultrasound evaluation of the hips according to the Graf technique. This was performed by a blinded single expert operator. Only one fetus was excluded from the analysis of the percentage distribution of the alpha and beta angle because of the suspicion of developmental dysplasia of the hip at prenatal ultrasound, confirmed postnatally.

### 2.4. Sample Dize

Owing to the central limit theorem, the size of the sample (*n* = 432) was large enough to build normal-based interval estimates of the study parameters.

### 2.5. Statistical Methods

Continuous variables were summarized as mean ± standard deviation; discrete and categorical variables were summarized as frequencies and percentages. The distribution of the alpha angle and the beta angle, overall and by gestational age, was illustrated using frequency polygons and percentile curves.

Inter-rater reliability of alpha-angle and beta-angle measurements made by the two operators was assessed with intra-class correlation coefficient (ICC) estimates and 95% confidence intervals (CIs) on the basis of a single-rating, absolute-agreement, two-way random-effects model [17]. Intra-rater reliability of measurements made by Operator 1 was assessed with ICC estimates and 95% CIs on the basis of a single-rating, absolute-agreement, two-way mixed-effects model. As a rule of thumb, values between 0.01 and 0.20 indicate “slight” agreement, values between 0.21 and 0.40 indicate “fair” agreement, values between 0.41 and 0.60 indicate “moderate” agreement, values between 0.61 and 0.80 indicate “substantial” agreement, and values between 0.81 and 1.00 indicate “almost perfect” agreement [18]. A correlation analysis was performed to evaluate the strength of the association between angles measured before and after birth.

All data were analyzed using the Stata 15 software (StataCorp. 2017. Stata Statistical Software: Release 15. College Station, TX, USA: StataCorp LP).

## 3. Results

In the study period, 433 uncomplicated term pregnancies underwent 3D ultrasound for the assessment of the fetal hip. One case was subsequently excluded because of confirmed postnatal diagnosis of developmental dysplasia of the hip. Table 1 shows the demographic and clinical characteristics of the final study sample of 432 cases. In 260 cases (60.2%), it was possible to insonate both fetal hips without adding extra scanning time. The number of left and right hips included is shown in Table 1. All volumes acquired were of good quality, yet for the purpose of this reproducibility study, we selected only one 3D volume for each fetus, irrespective of laterality.

Alpha and beta Graf angles were measured offline in the multiplanar mode by two experienced operators.

**Figure 2:**
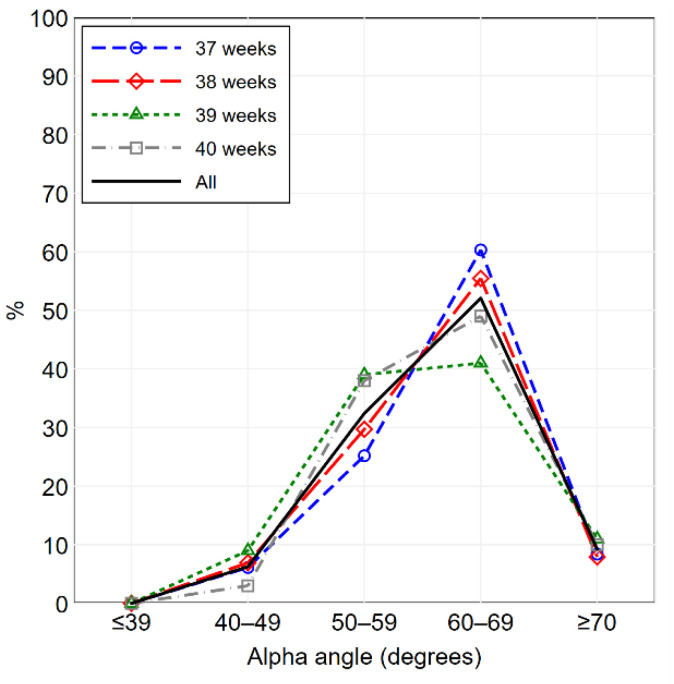
Alpha-angle frequency polygon, overall and by gestational age (37 weeks: *n* = 131; 38 weeks: *n* = 101; 39 weeks: *n* = 100; 40 weeks: *n* = 100).

**Figure 3:**
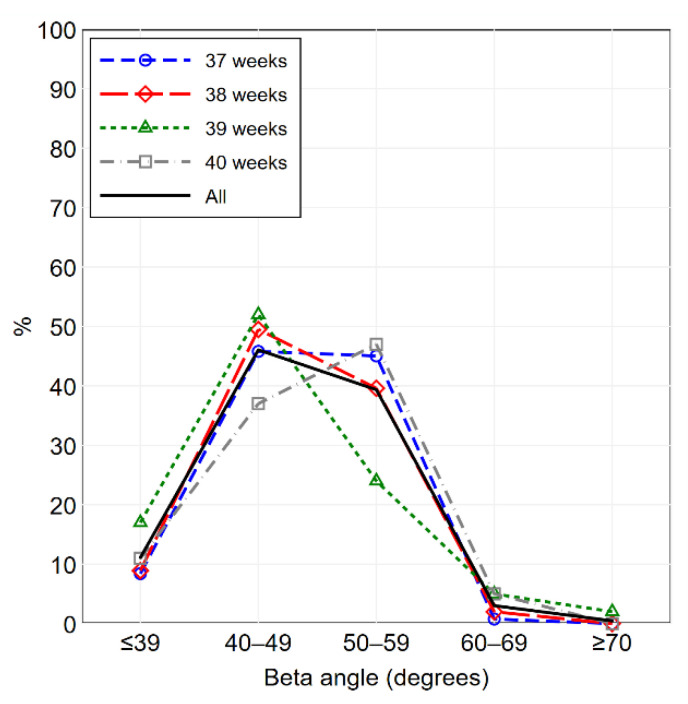
Beta-angle frequency polygon, overall and by gestational age (37 weeks: *n* = 131; 38 weeks: *n* = 101; 39 weeks: *n* = 100; 40 weeks: *n* = 100).

**Figure 4:**
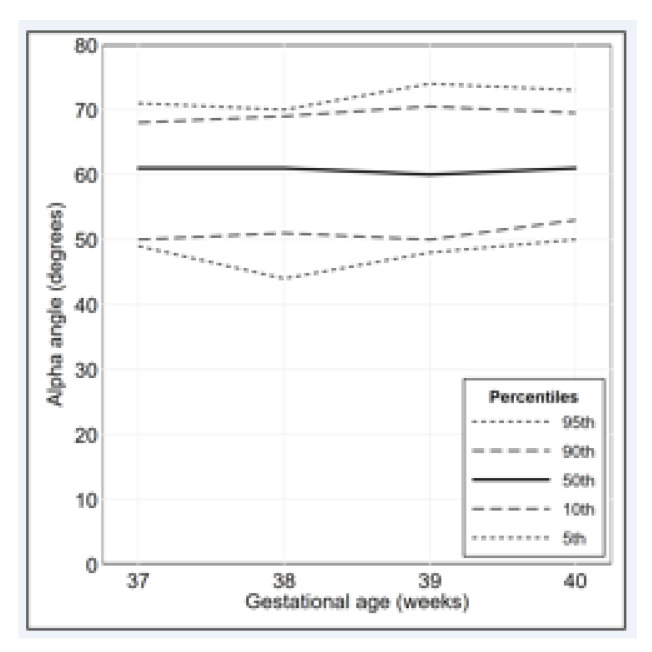
Alpha-angle percentile curves for gestational age 37 to 40 weeks (37 weeks: *n* = 131; 38 weeks: *n* = 101; 39 weeks: *n* = 100; 40 weeks: *n* = 100).

**Figure 5:**
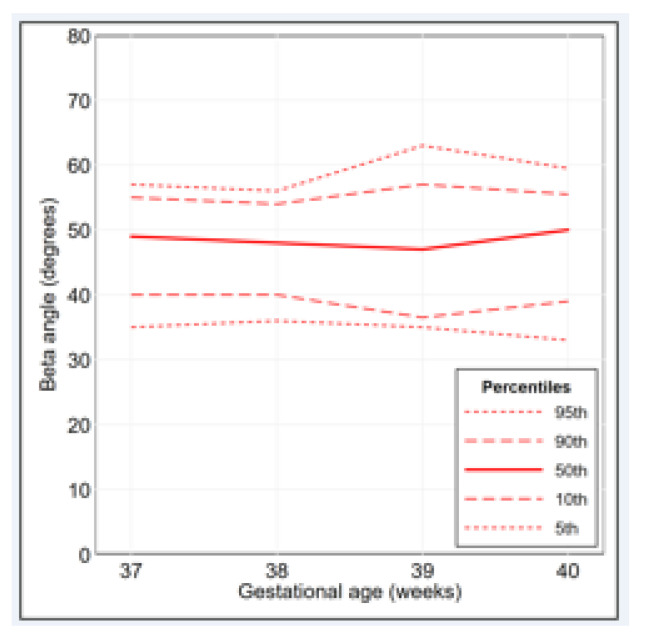
Beta-angle percentile curves for gestational age 37 to 40 weeks (37 weeks: *n* = 131; 38 weeks: *n* = 101; 39 weeks: *n* = 100; 40 weeks: *n* = 100).

The percentage distributions of the alpha angle and the beta angle are illustrated in Figure 2 and Figure 3, respectively. The percentile curves that illustrate the distribution of the alpha angle and the beta angle for gestational age are shown in Figure 4 and Figure 5, respectively. The values of alpha-angle and beta-angle percentiles, as well as the post-natal angles, are given in Table 2. As shown in Table 3 and Table 4, the inter-rater and intra-rater reliabilities of both angles were substantial. Most alpha angles lay between 60 and 69 degrees in all gestational ages, while most beta angles lay between 40 and 59 degrees. Ten fetuses (2.3%) had an alpha angle < 60 degrees and a beta angle > 55 degrees.

**Figure 6:**
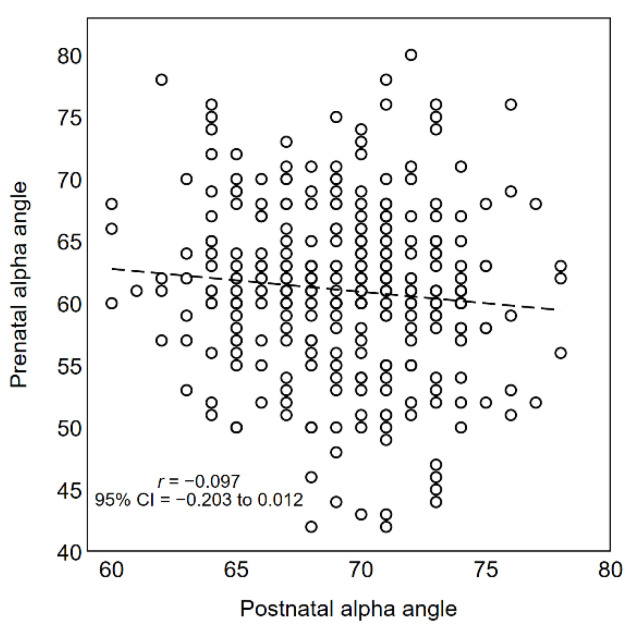
Scatter plot of alpha angles measured before and after birth (*n* = 324). Dashed line indicates the linear correlation between the two measurements.

**Figure 7:**
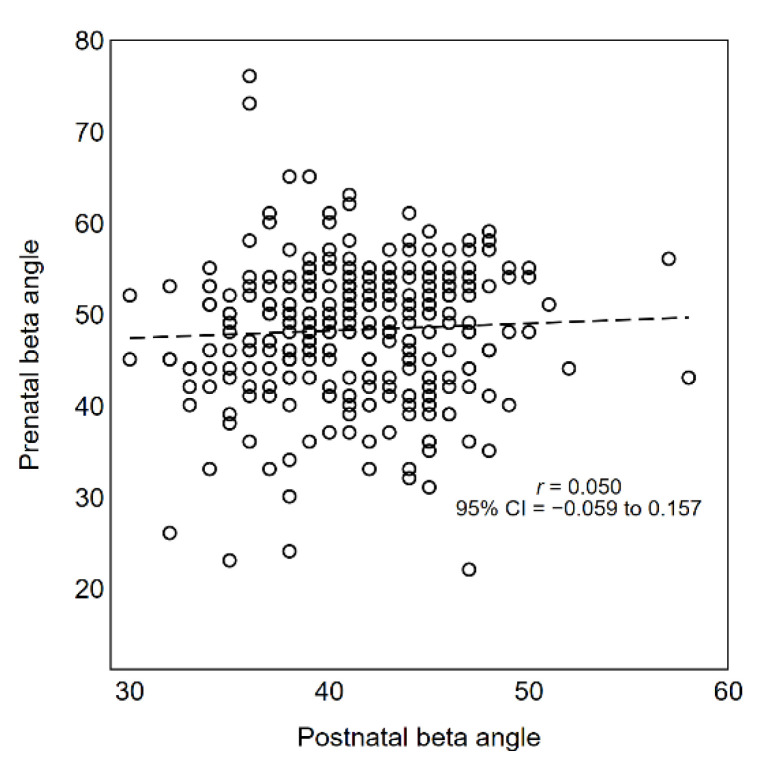
Scatter plot of beta angles measured before and after birth (*n* = 324). Dashed line indicates the linear correlation between the two measurements.

As shown in Figure 6 and Figure 7, there was no evidence of a linear relationship between prenatal and postnatal alpha angles (Pearson *r* = −0.097; 95% CI −0.203 to 0.012) and beta angles (Pearson *r* = 0.050; 95% CI −0.059 to 0.157). However, in the postnatal evaluation, alpha angles were systematically larger and beta angles systematically smaller.

## 4. Discussion

### 4.1. Principal Findings

Developmental dysplasia of the hip is an important clinical problem, affecting infants and families worldwide. As this condition is the most common pediatric hip disorder causing considerable long-term debilitation if left untreated, a prenatal diagnosis of this condition would be useful, especially in settings where a postnatal universal screening is not available. Indeed, in most countries, only a targeted screening is routinely guaranteed, with the concrete possibility of missing some cases.

The evaluation of the coxofemoral joint in fetuses at term of gestation has never been attempted before. The Graf technique application, currently employed at postnatal ultrasound, may be adapted to prenatal ultrasound with a substantial reproducibility.

### 4.2. Results

On the basis of our results, measuring these two angles at ultrasound appears feasible. The measurements obtained for both alpha and beta angles appeared fairly constant with a narrow interval of values. The intra-observer and inter-observer agreement for both seemed very good, with high intra-class correlation coefficient (ICC) and very small 95% CI.

There was no linear relationship between prenatal and postnatal alpha angles and beta angles. However, in the postnatal evaluation, alpha angles were systematically larger and beta angles systematically smaller; this could be explained by the physiological immaturity of the fetal hip before birth.

### 4.3. Clinical Implication

These results may have useful implications in clinical practice. Indeed, our measurement of alpha and beta angles may now be looked at as a reliable reference when assessing the normal anatomy of the coxofemoral joint in fetuses at term of gestation. We think that looking for the y-sign in fetuses at term is easy and not time-consuming—this could be done at routine ultrasound and does not require a more trained staff member. In this scenario, some more post-natal examinations would be performed, due to a prenatal suspicion of developmental dysplasia of the hip, but this is a little price to pay in order to reduce the long-term disabilities deriving from this undiagnosed condition.

### 4.4. Research Implication

Further research is needed to establish if developmental dysplasia of the hip could be diagnosed antenatally using our modified Graf technique.

### 4.5. Strengths and Limitations

To our knowledge, no study has ever attempted to evaluate the coxofemoral joint in a large number of fetuses. Moreover, our study provides reference ranges for fetal ultrasound hip measurements. The sonographic evaluation of coxofemoral joint in the prenatal period is feasible, with a technique that is easy to learn and not time-consuming. Indeed, the Graf angles can be easily measured at traditional 2D ultrasound. For the purpose of this study, a reproducibility study, we used 3D ultrasound in the multiplanar mode to measure alpha and beta angles, as this technique provides many advantages. Firstly, it permits a simultaneous visualization of the same structure in the three planes, making it easier to locate it in the space and to obtain a correct alignment of the structures involved in the angle measurements. Moreover, the enhancement volume contrast imaging mode now allows for the achievement of excellent quality images, often even better than those obtained with 2D ultrasound. Finally, it takes only a few seconds to acquire a good volume dataset, with the analysis of the angles performed offline by two independent operators measuring the same angles on the same volumes.

A strength of our study is that the fetal position in the uterus is the same as the lateral decubitus position that newborns have to adopt during the postnatal acquisition (hips flexed at 30 to 45°). Moreover, the ultrasound acquisition of the Graf angles in fetuses is easy because fetuses near term are static.

A limitation of our study could be that, in about 40% of our cases, we acquired only the proximal coxofemoral joint due to unfavorable fetal position. However, the purpose of the present study was to collect a large number of normal fetal hip 3D volumes in order to test the reproducibility of our modified Graf technique and to build reference range curves for normal fetuses at term, rather than spotting pathological cases. Further research is needed to establish if this technique might be used as an antenatal screening for developmental dysplasia of the hip; if this will be the case, a second scan might be arranged if it is not possible to acquire both junctions at the same time.

Another possible limitation of our study is that it is not clear whether the maturity of the fetal hip is complete near term of gestation. According to Bialik et al. in 1999 [1], 90% of newborn hips with clinical or sonographic signs of developmental dysplasia of the hip improved spontaneously before two to six weeks of age, probably because of a normal immaturity of hip development. On the contrary, the opinion of Stiegler et al., on the basis of an ultrasonographic study of the prenatal hip joints, is that the fetal hip joint is sonographically mature at 34 weeks of gestation [19]. However, the role of antenatal screening might be identifying those fetuses at risk of developing this condition in order to arrange a dedicated postnatal scan, rather than diagnosing it before birth. In our cohort, we had one case of developmental dysplasia of the hip that was suspected at antenatal ultrasound and then confirmed postnatally. No other case of developmental dysplasia of the hip was detected at postnatal ultrasound. An ongoing prospective study in our center aims at establishing if antenatal ultrasound is reliable in identifying pathological cases.

## 5. Conclusions

The evaluation of the coxofemoral joint in fetuses at term of gestation has never been attempted before. The Graf technique application, currently employed at postnatal ultrasound, may also be adapted to prenatal ultrasound with a substantial reproducibility, even if there was no evidence of a linear relationship between prenatal and postnatal alpha angles and beta angles. Further research is needed to establish if this method allows for an accurate diagnosis of developmental dysplasia of the hip in utero. However, if the evaluation of the fetal hips became part of routine ultrasound assessment in the third trimester, this could improve the possibility of diagnosis of developmental dysplasia of the hip, either directly or through a targeted postnatal screening for suspected cases. In this scenario, both fetal hips should be systematically analyzed.

## Figures and Tables

**Table 1:** Demographic and clinical characteristics of the study sample (*n* = 432).

Characteristics	*n* (%)
Age at evaluation (years), mean ± SD	33.7 ± 5.1
Ethnicity	
Caucasian	391 (90.5)
African	22 (5.1)
Asian	19 (4.4)
Family history of DDH	16 (4.4)
Other offspring with DDH	1 (0.2)
Twin pregnancy	0 (0.0)
Presentation	
Vertex	392 (90.7)
Breech	40 (9.3)
Multiparous/primiparous	183 (42.4)
Right hips	235 (54.4)
Left hips	197 (45.6)

**Table 2:** Values of alpha-angle and beta-angle percentiles for gestational age 37 to 40 weeks (37 weeks: *n* = 131; 38 weeks: *n* = 101; 39 weeks: *n* =100; 40 weeks: *n* = 100). Post-natal percentiles are also presented (*n* = 324).

Gestational Age (Weeks)	Alpha Angle (Degrees)					Beta Angle (Degrees)				
	5th	10th	50th	90th	95th	5th	10th	50th	90th	95th
37	49	50	61	68	71	35	40	49	55	57
38	44	51	61	69	70	36	40	48	54	56
39	48	50	60	70.5	74	35	36.5	47	57	63
40	50	53	61	69.5	73	33	39	50	55.5	59.5
After birth	64	65	70	74	74	34	36	41	47	48

**Table 3:** Inter-rater reliability of the alpha angle and the beta angle for developmental displacement of the hip evaluation.

Angle	ICC	95% Confidence Interval of ICC	
		Lower Boundary	Upper Boundary
Alpha	0.702	0.650	0.748
Beta	0.737	0.691	0.778

**Table 4:** Intra-rater reliability of the alpha angle and the beta angle for developmental displacement of the hip evaluation.

Angle	ICC	95% Confidence Interval of ICC	
		Lower Boundary	Upper Boundary
Alpha	0.692	0.639	0.738
Beta	0.750	0.706	0.789

## Data Availability

The data presented in this study are available on request from the corresponding author. The data are not publicly available due to privacy.
